# Supplementary material for: Reduced Context Updating but Intact Visual Priors in Autism
Source: Comput Psychiatr. 2021 Dec 29;5(1):140–58. doi: 10.5334/cpsy.69 (PMC11104295; doi:10.5334/cpsy.69)
Supplement: Supplementary materials. [file cpsy-5-1-69-s1.pdf]

Supplementary Material

1.0 Group Results

We conducted 2x2x2 repeated measures analysis of variance for each measure of interest, with prior (Narrow vs Wide) and Likelihood (Narrow vs Wide) and Group (AS vs NT) as Factors. We also report independent samples t-test comparisons for individual conditions as well.

F- Statistic (F), Effect size (partial eta^2 / η_p^2), Bayes Factor with evidence for the null (BF_{01}), t-statistic (t) and p-values (p) are shown.

1.1 2-Prior Task: Likelihood Reliance (i.e., sensory weight)

Table S1: Independent samples comparison of sensory weights by condition

Outcome Variables	Group	Mean *(M)*	Bayes Factor (BF_{01})	*t-statistic*	*p*-value
$P_N L_N$	NT	0.610	1.975	1.407	0.166
	AS	0.714			
$P_N L_W$	NT	0.521	2.747	1.108	0.274
	AS	0.601			
$P_W L_N$	NT	0.857	4.241	-0.499	0.620
	AS	0.840			
$P_W L_W$	NT	0.769	0.921	-1.939	0.058
	AS	0.666			

1.2 2-Prior Task: Performance (Estimation Error)

Table S2: Results from repeated-measures ANOVA for estimation error

Estimation Error	*F-statistic*	Effect Size (η_p^2)	*p*-value
Main Effect Group	1.206	0.025	0.278
Group*Prior	1.772	0.036	0.189
Group*Likelihood	2.306	0.046	0.135
Group*Prior*Likelihood	0.971	0.020	0.329

Table S3: Between group effects for estimation error

Outcome Variables	Group	M	BF_{01}	*t*	*p*
$P_N L_N$	NT	0.026	3.574	0.795	0.430
	AS	0.028			
$P_N L_W$	NT	0.045	1.486	1.624	0.111
	AS	0.053			
$P_W L_N$	NT	0.033	4.739	0.045	0.965
	AS	0.033			
$P_W L_W$	NT	0.060	3.372	0.874	0.387
	AS	0.062			

1.3 2-Prior Task: Subjective Prior Variance (calculated with true likelihood as set by the task)

Table S4: Results from repeated-measures ANOVA for prior variance

Estimation Error	*F*	η_p^2	*p*
Main Effect Group	1.355	0.029	0.250
Group*Prior	0.372	0.008	0.545
Group*Likelihood	1.888	0	0.887
Group*Prior*Likelihood	3.914	0.078	0.054

Table S5: Between group effects on the subjective prior variance

Outcome Variables	Group	*M*	BF_{01}	*t*	*p*
P_NL_N	NT	0.004	4.455	-0.374	0.969
	AS	0.003			
P_NL_W	NT	0.009	2.544	1.184	0.250
	AS	0.025			
P_WL_N	NT	0.010	2.292	1.273	0.163
	AS	0.072			
P_WL_W	NT	0.039	3.158	-0.945	0.314
	AS	0.022			

1.4 2-Prior Task: Confidence

Table S6: Results from repeated-measures ANOVA for confidence

Estimation Error	*F*	η_p^2	*p*
Main Effect Group	2.381	0.047	0.129
Group*Prior	2.200	0.044	0.145
Group*Likelihood	9.172	0.160	0.004*
Group*Prior*Likelihood	0.552	0.011	0.461

Table S7: Between group effects on confidence

Outcome Variables	Group	*M*	BF_{01}	*t*	*p*
P_NL_N	NT	54.274	0.620	-2.170	0.035*
	AS	42.713			
P_NL_W	NT	48.889	2.012	-1.392	0.170

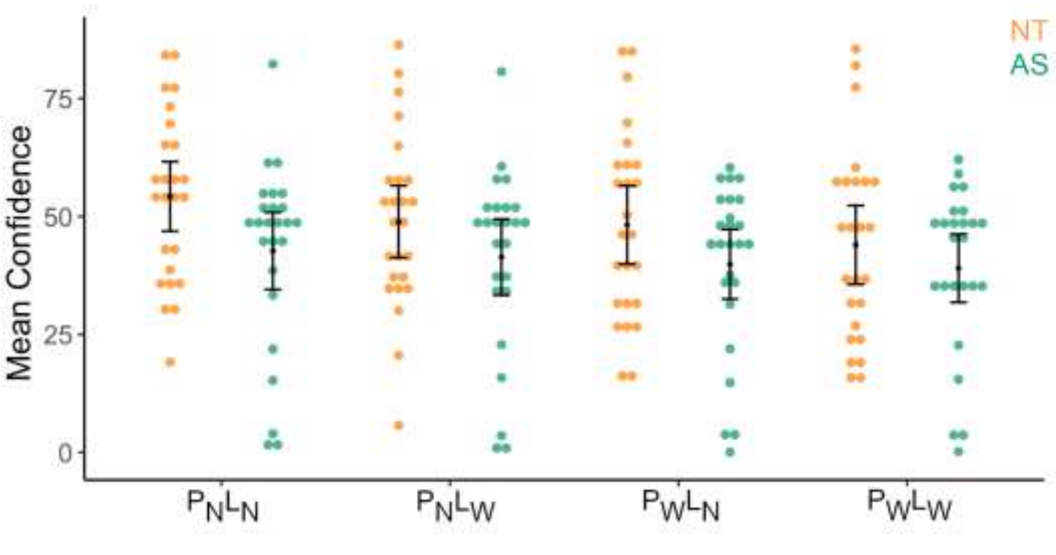

	AS	41.388			
$P_W L_N$	NT	48.217	1.655	-1.546	0.129
	AS	39.898			
$P_W L_W$	NT	43.984	3.251	-0.920	0.362
	AS	39.048			

1.5 2-Prior Task: Trial-by-trial sensory weights

Table S8: Results from repeated-measures ANOVA for trial-by-trial sensory weights

**Estimation Error**	***F***	**η_p^2**	***p***
Main Effect Group	4.381	0.084	0.042*
Group*Prior	2.765	0.054	0.103
Group*Likelihood	2.829	0.056	0.099
Group*Prior*Likelihood	3.405	0.066	0.071

Table S9: Between group effects for trial-by-trial sensory weights

**Outcome Variables**	**Group**	***M***	**BF_{01}**	***t***	***p***
$P_N L_N$	NT	0.056	0.798	2.026	0.048
	AS	0.068			
$P_N L_W$	NT	0.042	0.966	1.910	0.062
	AS	0.055			
$P_W L_N$	NT	0.021	4.222	0.510	0.613
	AS	0.022			
$P_W L_W$	NT	0.040	0.380	2.429	0.019*
	AS	0.050			

2.0 Continuum Analysis – Autism Traits

We undertook Spearman **correlation analysis** for each research question.

2.1 Main Task:

Table S10: Spearman correlations with Autism Quotient in the main 2-prior task

AQ Score correlated with:	r_S	*p*	**95% CI**	
**Likelihood Reliance (Slopes)**				
$P_N L_N$	0.042	0.714	-0.180	0.266
$P_N L_W$	0.038	0.740	-0.185	0.271
$P_W L_N$	0.024	0.834	-0.191	0.245
$P_W L_W$	-0.040	0.724	-0.270	0.200
**Estimation Error**				
$P_N L_N$	-0.244	0.030*	-0.444	-0.017
$P_N L_W$	-0.094	0.411	-0.302	0.133
$P_W L_N$	-0.171	0.133	-0.371	0.041
$P_W L_W$	-0.234	0.038*	-0.442	-0.002
**Subjective Prior (with objective Likelihood)**				
$P_N L_N$	-0.015	0.900	-0.242	0.231
$P_N L_W$	0.059	0.612	-0.160	0.291
$P_W L_N$	0.095	0.410	-0.155	0.325
$P_W L_W$	0.017	0.882	-0.204	0.247
**Trial-by-Trial Sensory Weights**				
$P_N L_N$	-0.183	0.103	-0.380	0.044
$P_N L_W$	-0.141	0.213	-0.348	0.106
$P_W L_N$	-0.260	0.020*	-0.443	-0.060
$P_W L_W$	-0.041	0.716	-0.252	0.185

2.2 Likelihood Only Task:

Spearman rank correlations with AQ scores. For the Likelihood Only Task we excluded outliers based on Tukey's 1.5 Inter-Quartile Range. Thus 7 participants (1NT and 6AS) were excluded from this analysis.

Table S11: Spearman correlations with Autism Quotient in the Likelihood Only Task

	NT (N=46)				AS (17)				All (N=73)			
AQ Score correlated with:	**r_S**	***p***	**95% CI**		**r_S**	***p***	**95% CI**		**r_S**	***p***	**95% CI**	
1. Accuracy (mean Estimation Error)												
Narrow	-0.283	0.057	-0.540	-0.001	-0.225	0.386	-0.724	0.355	-0.254	0.038	-0.487	-0.004
Wide	-0.363	0.013 (0.026)*	-0.621	-0.094	-0.158	0.560	-0.665	0.412	-0.359	0.003 (0.012) *	-0.565	-0.125
2. Variance of Estimation Error (i.e., Likelihood Variance)												
Narrow	-0.051	0.736	-0.307	0.229	0.004	0.987	-0.575	0.557	0.062	0.618	-0.175	0.289
Wide	-0.303	0.041 (0.081)	-0.588	-0.017	0.306	0.249	-0.232	0.804	-0.326	0.007 (0.028) *	-0.542	-0.082

3.0 Group by Autism Traits Interaction Effects

We conducted Multivariate analysis of covariance with AQ as covariate, Group as fixed factor and variable interest as the outcome variable(s) to assess the *interaction between group and AQ*.

3.1 Likelihood Only Task:

For the Likelihood Only Task we excluded significant outliers based on Tukey's 1.5 Inter-Quartile Range of sensory weights in the Likelihood Only task. Thus 7 participants (1NT and 6AS) were excluded from the analysis. Thus, the total sample is 73 participants.

Table S12: ANCOVA

	Outcome variable		**F**	**p-value**
1.	Mean Estimation Error	Narrow Likelihood	1.757	0.165
		Wide Likelihood	3.503	0.020*
2.	Variance of Estimation Error	Narrow Likelihood	0.435	0.728
		Wide Likelihood	3.107	0.033*

3.2 Main / 2-Prior Task:

Table S13: ANCOVA

	Outcome variable		**F**	**p-value**
1.	Sensory Weight	$P_N L_N$	1.539	0.211
		$P_N L_W$	1.223	0.307
		$P_W L_N$	0.230	0.875
		$P_W L_W$	0.978	0.408
2.	Subjective Prior	$P_N L_N$	0.205	0.892
		$P_N L_W$	0.816	0.489
		$P_W L_N$	2.305	0.084
		$P_W L_W$	0.575	0.633
3.	Mean Estimation Error	$P_N L_N$	2.682	0.053
		$P_N L_W$	3.350	0.023*
		$P_W L_N$	1.578	0.202
		$P_W L_W$	7.893	1.19×10^{-4}*
3.	Mean Confidence	$P_N L_N$	4.337	0.007*
		$P_N L_W$	3.870	0.012*
		$P_W L_N$	5.202	0.003*
		$P_W L_W$	4.217	0.008*

4.0 Autism Subscale Analysis – Attention to detail

Table S14: Spearman correlations with AQ subscale attention to detail

AQ Attention to Detail correlated with:	r_S	p-value	95% CI	
Likelihood Only Task:				
1. Likelihood Variance (RE)				
Narrow	-0.004	0.974	-0.239	0.224
Wide	-0.375	0.002*	-0.555	-0.147
2. Estimation Error				
Narrow	-0.256	0.036	-0.480	-0.019
Wide	-0.359	0.003*	-0.532	-0.143
3. Sensory Weight				
Narrow	-0.130	0.296	-0.369	0.131
Wide	-0.005	0.968	-0.265	0.251

5.0 Dimensional Analysis – Sensory Sensitivities

Spearman rank correlations with SPQ scores

5.1 Main Task:

Table S15: Spearman correlations with SPQ scores in the 2-Prior Task

	r_S	*p*	95% CI	
1. Likelihood Reliance (Slopes)				
P_NL_N	-0.091	0.425	-0.311	0.154
P_NL_W	-0.075	0.511	-0.305	0.162
P_WL_N	-0.196	0.083	-0.412	0.029
P_WL_W	-0.109	0.338	-0.321	0.126
2. Estimation Error				
P_NL_N	0.116	0.309	-0.124	0.345
P_NL_W	0.017	0.885	-0.227	0.250
P_WL_N	0.173	0.128	-0.052	0.391
P_WL_W	0.104	0.363	-0.141	0.317
3. Prior Variance				
P_NL_N	0.070	0.548	-0.161	0.278
P_NL_W	-0.090	0.434	-0.314	0.136
P_WL_N	-0.217	0.058	-0.420	0.005
P_WL_W	-0.155	0.178	-0.365	0.063
3. Trial-by-trial sensory weights				
P_NL_N	0.057	0.616	-0.167	0.286
P_NL_W	0.009	0.937	-0.199	0.235
P_WL_N	0.018	0.877	-0.203	0.248
P_WL_W	-0.041	0.716	-0.252	0.185

5.2 Likelihood Only Task

Table S16: Spearman correlations with SPQ scores in the Likelihood Only Task

SPQ Score correlated with:	r_S	*p*	95% CI	
1. Variance of Estimation Error				
Narrow	-0.046	0.696	-0.197	0.279
Wide	0.097	0.409	-0.132	0.314
2. Mean Estimation Error				
Narrow	0.001	0.996	-0.254	0.238
Wide	0.102	0.389	-0.131	0.322

6.0 ADOS Scores

Bootstrapped Spearman rank correlations with ADOS scores

Table S17: ADOS Scores and behavioural measures

ADOS Score correlated with:	r_S	p-value	95% CI	
Likelihood Only Task:				
4. Likelihood Variance (RE)				
Narrow	0.080	0.687	-0.326	0.447
Wide	0.099	0.616	-0.336	0.489
5. Estimation Error				
Narrow	0.186	0.353	-0.247	0.581
Wide	0.105	0.602	-0.329	0.529
6. Likelihood Reliance				
Narrow	0.098	0.626	-0.363	0.563
Wide	-0.164	0.413	-0.516	0.263
2-Prior Task:				
1. Subjective prior variance				
$P_N L_N$	0.079	0.674	-0.267	0.405
$P_N L_W$	-0.014	0.939	-0.375	0.333
$P_W L_N$	-0.277	0.131	-0.626	0.143
$P_W L_W$	-0.266	0.148	-0.567	0.135
2. Estimation Error				
$P_N L_N$	0.256	0.164	-0.138	0.621
$P_N L_W$	0.186	0.317	-0.178	0.524

$P_W L_N$	0.199	0.282	-0.214	0.545
$P_W L_W$	0.284	0.122	-0.110	0.630

7.0 Regression model fit tests

We investigated if the regression model fit was significant for each condition and each participant. We find the regression model was significant ($p<0.5$) for each condition for most participants.

However, 9 (out of 80) participants showed non-significant p-values ($p<0.05$) in one or two conditions but were significant for the other conditions. (These 9 participants included 5 'NT', 4 'AS' and 2 'Other'). We have not excluded these for any analysis.

2 subjects in the AS group (i.e., with a confirmed diagnosis of ASD) however, showed non-significant p-values for all four conditions. These participant's parameter estimates are not outliers amongst other participants. However, we have conducted the group analysis that impacts our major conclusions excluding these two participants and found similar results, hence we decided to keep the original results with all participants included in the main text. Below are the results excluding the two participants from group analysis:

1) Likelihood reliance in the 2-prior task

   We found evidence for no difference between the groups in overall likelihood reliance, (i.e., sensory weights averaged across conditions) [t = -0.448, BF_{01} = 4.256, p = 0.656].

2) Context adjustment in the 2-prior task

   Looking within conditions in the 2-Prior Task, a repeated-measures ANOVA (see methods) of sensory weights revealed a significant Group*Prior [Effect Size $\eta_p^2 = 0.109$, p = 0.022], a non-significant trend in Group*Likelihood [$\eta_p^2 = 0.067$, p = 0.071] interaction, but no Group*Prior*Likelihood [$\eta_p^2 = 0.027$, p = 0.260] interaction.
